# Screening of Corrosion in Storage Tank Walls and Bottoms Using an Array of Guided Wave Magnetostrictive Transducers

**DOI:** 10.3390/s26041253

**Published:** 2026-02-14

**Authors:** Sergey Vinogradov, Nikolay Akimov, Adam Cobb, Jay Fisher

**Affiliations:** Sensor Systems and NDE Technology Section, Structural Engineering Department, Southwest Research Institute, San Antonio, TX 78238, USA; nikolay.akimov@swri.org (N.A.); adam.cobb@swri.org (A.C.); jay.fisher@swri.org (J.F.)

**Keywords:** magnetostrictive transducer arrays, guided wave testing, storage tank inspection, full matrix capture, total focusing method

## Abstract

Aboveground storage tanks are used to store various fluids and chemicals for many industrial purposes. According to API standard 653, the structural integrity of these tanks must be regularly assessed. The U.S. EPA requires each operator to have a Spill Prevention, Control and Countermeasure Plan (SPCC) for aboveground storage containers. The accepted practice for inspection of these tanks, particularly the tank bottoms, requires removing the tank from service, emptying the tank, and interior entry for direct inspection of the structure. The required inspection operations are hazardous due to the chemicals themselves as well as the requirement to operate within confined spaces. An inspection from outside the tank would have significant cost and time benefits and would provide a large reduction in the risks faced by inspection personnel. Guided wave (GW) testing is a promising candidate for screening of storage tank walls and bottoms from the tank exterior due to the ability of GWs to propagate over long distances from a fixed probe location. The lowest-order transverse-motion guided wave modes (e.g., torsional vibrations in pipes) are a good choice for long-range inspection because this mode is not dispersive; therefore, the wave packets do not spread out in time. A common weakness of guided wave inspection is the complexity of report generation in the presence of multiple geometry features in the structure, such as welds, welded plate corners, attachments and so on. In some cases, these features cause generation of non-relevant indications caused by mode conversion. Another significant challenge in applying GW testing is development of probes with high-enough signal amplitudes and relatively small footprints to allow them to be mounted on short tank bottom extensions. In this paper, a new generation of magnetostrictive transducers will be presented. The transducers are based on the reversed Wiedemann effect and can generate shear horizontal mode guided waves over a wide frequency range (20–150 kHz) with SNRs in excess of 50 dB. The recently developed SwRI MST 8 × 8 probe contains an array of eight pairs of individual magnetostrictive transducers (MsTs). The data acquisition hardware allows acquisition using Full Matrix Capture (FMC) and analysis software reporting of anomalies based on Total Focusing Method (TFM) image reconstruction. This novel inspection package allows generation of reports that map out corrosion locations and provide estimates of defect widths. Case studies of this technology on actual storage tank walls and bottoms will be presented together with validation of processing methods on mockups with known anomalies and geometry features.

## 1. Introduction

Typical industrial storage tanks range in diameter from 2.5 m to 100 m. There is a great variety of tank bottom geometries (lap-welded, butt-welded or both). Guided wave methods for inspecting the tank floor from the exterior have been investigated by different research groups for at least a decade. In one case, an array of piezoelectric transducers placed around the annular skirt plate was used to receive a tomographic image of a lap-welded tank bottom condition in [1]. The attenuation profile of 50 kHz lowest-order symmetric (S0) mode guided waves was used to reconstruct an 8 m diameter tank bottom condition, but the accuracy limits were not determined. Other research indicated that a similar tomographic approach could be utilized for finding 100 mm diameter 50% deep anomalies. The researchers placed 48 transducers around a 4.1 m diameter tank bottom mockup [2]. The use of a magnetostrictive guided wave probe in pulse-echo configuration to scan the tank floor by moving the probe along the outside skirt plate was reported in [3]. Wall thinning and pit holes were successfully detected with the help of synthetic aperture focusing technique (SAFT) processing. Anomalies were detected in the annular ring area (before the first lap weld). A method utilizing higher-order mode cluster (HOMC) guided waves for online defect detection in the annular plate region of aboveground storage tanks was reported in [4]. The method is applicable for corrosion mapping in the region of one meter and below from the vertical wall with a probe positioned outside of the tank. Another method of anomaly detection in storage tank floors was based on placing guided wave probes on storage tank walls [5]. It was found that shear horizontal (SH) guided wave modes provided a superior signal-to-noise ratio compared to the S0 mode in the tank floor. Damage detection in hazardous waste storage tank bottoms using ultrasonic guided waves was reported in [6]. Detection was accomplished using an SH0 mode guided wave EMAT crawler in the frequency range of 45–64 kHz in a butt-welded tank bottom. Penetration capability of the S0 mode in lap-welded tank bottoms was reported in [7]. It was shown that guided waves in the frequency range of 20–40 kHz provided the best penetration. Bottom plate damage localization based on dual-array SH0 mode piezoelectric sensors installed on the tank bottom extension and also on the tank wall was reported in [8]. It was shown that utilizing the dual array helped to eliminate non-relevant indications produced by piezoelectric transducers.

The guided wave methods relevant to tank inspection can be split into two groups—methods targeting long-range coverage at the cost of lower sensitivity, and methods providing shorter-range coverage (mostly in the area before the first lap weld), but delivering more accurate defect information.

## 2. Magnetostrictive Sensors

**Figure 1:**
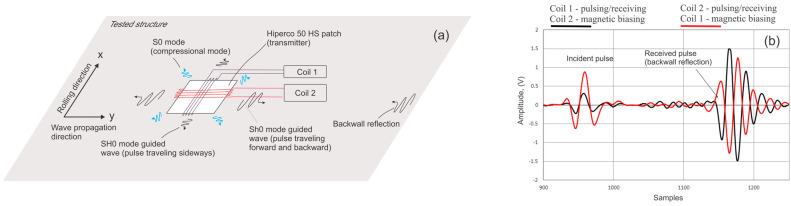
Generation of shear horizontal vibrations using a ferromagnetic patch: (**a**)—generated vibration modes with respect to patch rolling directions; (**b**)—signal generated in y direction by switching pulsing and magnetic biasing circuits.

The physical effect for generation of transverse vibration using orthogonally oriented static and variable magnetic fields was discovered by Wiedemann for generation of torsional motion in rod-shaped components [9]. In a similar evaluation on a plate, an SH wave transducer consisting of a meander coil and static bias magnetic field parallel to the coil elements was described by Thompson in 1979 [10]. In this configuration, the SH wave propagated in the direction perpendicular to the static magnetic bias direction. In an alternative design, the static and pulsed fields were reversed, and the SH wave propagated in the direction parallel to the static magnetic bias; this method was called the reversed Wiedemann effect [11,12]. For practical NDE, increased signal amplitudes are always a way to accomplish better coverage and sensitivity. This is why both effects have been implemented using a soft ferromagnetic patch material (typically nickel or FeCo alloy). The Hiperco 50 HS alloy, typically used for this purpose (48.94% iron, 48.75% cobalt, 1.90% vanadium, 0.30% niobium, 0.05% manganese, 0.05% silicon, and 0.01% carbon), is a soft magnetic alloy commercially available in the form of strips or rods of various dimensions. The magnetic properties of the alloy in DC fields have been well studied as a function of the annealing regime and rolling process and were summarized by Bozorth [13]. The alloy has a rather high magnetostrictive coefficient (about 60 × 10^−6^ versus 30 × 10^−6^ for nickel). It is mechanically strong (yield strength 40 to 60 kpsi versus less than 20 kpsi for nickel) and exhibits magnetic saturation at 24 kG. The alloy was successfully adopted for guided wave testing of pipelines in 2002 [14]. The performance of a FeCo patch in application to generation of shear horizontal guided waves in plate-like and cylindrical structures was further investigated in [15,16]. Figure 1a shows an example of such a patch attached to a tested structure. In this example, two mutually orthogonal coils are wrapped around the patch, one parallel to the patch rolling direction (Y direction) and another perpendicular to the rolling direction. To provide high magnetic permeability and lower coercivity, the patch was annealed in a furnace at 750 deg C for 30 min.

In this arrangement, one coil provides a time-varying magnetic field, and the other coil provides a permanent magnetic bias with duration 40 microseconds (to fully cover the excitation time of coil 1), with both coils generating in-plane magnetic fields. It was experimentally confirmed that as long as the wave propagates perpendicular to the patch rolling direction and the patch magnetization level produced by both coils is nearly equal (of the order of 10 kG), the signal amplitudes generated are similar regardless of which coil operates as the pulsing coil. As an example, Figure 1b shows the reflection from the plate end produced by a 25 × 25 mm FeCo patch with coil 1 pulsing and coil 2 providing magnetic bias (black trace) and coil 2 pulsing and coil 1 providing magnetic bias (red trace) at 60 kHz central frequency. This experiment explains why generation of SH0 modes traveling in the desired direction is always accompanied by SH0 modes traveling in the undesired direction.

The shear horizontal wave amplitude is influenced by the aspect ratio of the patch. For example, if direction Y is the desired propagation direction, making the patch longer in the X direction makes the signal amplitude propagating in the Y direction higher, and the sideways portion of energy (direction X) will be reduced.

**Figure 2:**
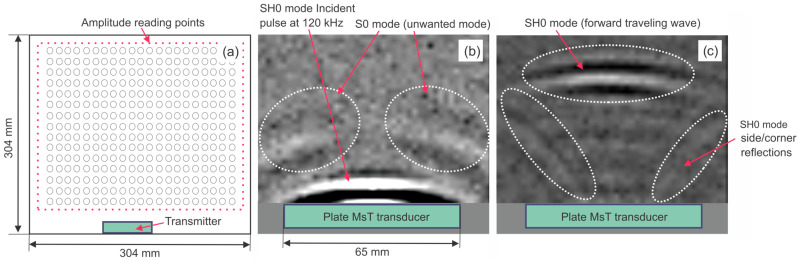
Results of vibration readings from a 304 × 304 mm aluminum plate with an SH0 mode probe coupled to one side of the plate: (**a**)—experiment arrangements; (**b**)—desired SH0 mode accompanied by undesired S0 mode; (**c**)—SH0 modes going sideways and creating additional waves.

In addition to SH0 modes propagating sideways, compressional S0 mode waves propagating through the 35–215° and 320–140° axes can be observed. This portion of energy is shown in Figure 1a with blue arrows. Figure 2 shows results of vibration readings from a 304 × 304 mm aluminum plate with an SH0 mode probe with a 65 mm aperture acoustically coupled to one side of the plate. It can be clearly seen in a that together with the wanted SH0 mode, an S0 mode is also generated as well as SH0 modes going sideways and creating additional waves (shown in Figure 2c). It should be noted that the patch can also detect the unwanted modes or energy propagating in the unwanted direction. This is why each particular probe design typically goes through a calibration process on mockups to characterize energy generation for desired and undesired modes and side effects specific to each probe geometry.

**Figure 3:**
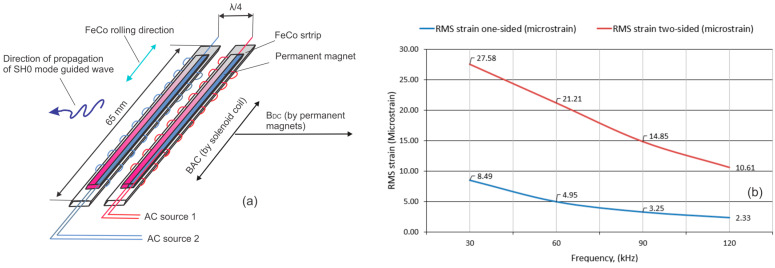
Conceptual design of reversed-Wiedemann-effect MsT probe: (**a**)—probe packaging; (**b**)—comparison of strains in strip mid-plane produced by two-sided coil versus one-sided coil.

Figure 3a shows the conceptual design of a reversed-Wiedemann-effect MsT probe. Major components of the MsT probe include two 0.18 mm thick, 12.7 mm wide and 76 mm long FeCo strips installed next to each other for applying direction control to the guided wave pulse. The probe is elongated in the rolled direction of the FeCo strip to reduce generation of unwanted wave modes. A coil wound around each strip provides the pulsed field, and a belt of small rare-earth magnets is placed on top of the strip to achieve uniform and self-sustained biasing magnetization in the wave propagation direction. The coil provides two-sided strip excitation. Magnetostrictive sensor designs relying on one-sided excitation will primarily activate near-surface regions opposite the tested structure, whereas symmetric (two-sided) excitation will excite the strip surface directly facing the tested structure. In addition, two-sided excitation significantly improves field penetration and the magnetostrictive effect in the mid-plane of the strip. As an example, Figure 3b shows calculated dependence of microstrain generated in the mid-plane of the 0.18 mm thick FeCo patch at 30, 60, 90 and 120 kHz test frequencies using one- and two-sided coils. The calculations were done with near-surface B equal to 15,800 G, FeCo resistivity ρ ≈ (3–5) × 10^−7^ Ω⋅m and FeCo permeability μ_r_ = 200. As can be seen from the plot, the two-sided coil produced 3.2 times greater strain at 30 kHz and 4.5 times greater strain at 120 kHz.

The entire sensor assembly is encapsulated into a flexible urethane coating to protect it from the environment. The effect of the thickness of the adhesive layer between the probe and tested structure on SH0 mode energy transfer was studied in [17]. It was found that peak performance could be achieved at 0.3 mm layer thickness, and moderate performance was supported at 0 and 0.5 mm layer thicknesses. In practical MsT designs, a 0.5 mm thick bonded fiberglass board is used to protect the AC coil from mechanical impact. The probe is sized to fit on tank bottom extensions as short as 20 mm.

## 3. Magnetostrictive Array Probes

**Figure 4:**
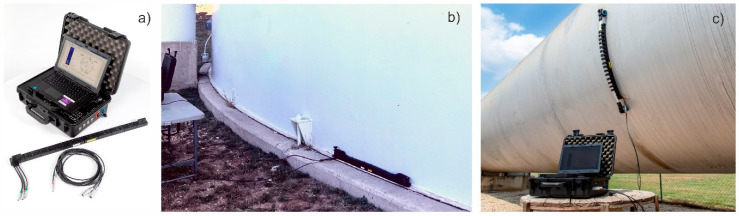
Magnetostrictive array system: (**a**)—MsSRV5M instrument with MsT 8 × 8-array probe; (**b**)—8 × 8 probe installed on storage tank bottom extension; (**c**)—probe installed on wall of large storage tank.

The next step in technology evolution is based on an array of sensors and advanced data collection and postprocessing algorithms. Figure 4 shows an MsT 8 × 8 probe with eight MsT segments constructed as described above. The probe has flexible joints between segments that let it be bent around a large radius in any direction. Each segment is individually connected to an SwRI MsSRV5M guided wave instrument with an integrated multiplexer. This instrument provides pitch-catch and pulse-echo data collection methods from every possible combination of sensor segments. The probe can be installed on the tank bottom extension or tank wall using a magnetic clamping fixture. Figure 4a shows the field-deployable MsSRV5M instrument with an MsT 8 × 8-array probe. Figure 4b,c show the probe installed on the tank bottom extension and on the wall of a large storage tank. A water-soluble shear wave couplant is used to facilitate acoustic coupling of the probe.

## 4. Data Acquisition and Image Reconstruction

Three different methods of data collection can be used with the 8 × 8 probe:

Synthetic aperture focusing technique (SAFT): each segment used as a pulser/receiver pair;

Common source method (CSM): all segments pulsed simultaneously, and each segment receives;

Full Matrix Capture (FMC) mode (TFM processing): all possible segment combinations are used for pulsing and receiving.

**Figure 5:**
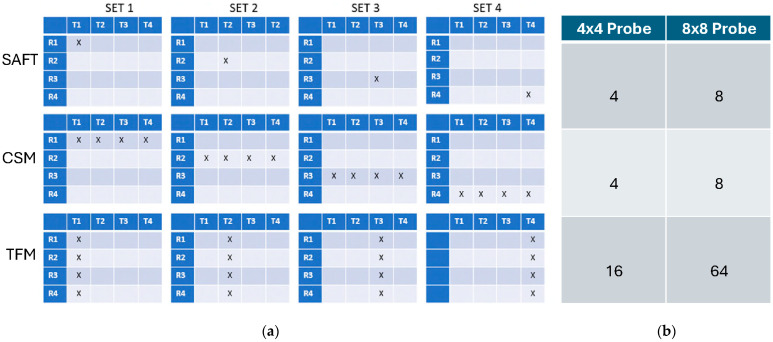
Data acquisition protocols used with 8 × 8-array MsT probe: (**a**)—segments engaged when acquiring data for SAFT, CSM, and TFM algorithms; (**b**)—number of datasets collected for each algorithm when using 4 × 4-array probe and 8 × 8 array.

Figure 5 illustrates the available data acquisition protocols and the number of datasets collected in each case.

The data acquisition time will vary depending on the acquisition method selected. For the SAFT and CSM, data collection of eight waveforms in both directions typically takes approximately 20 sec. For the TFM (FMC collection mode), average acquisition time is about 3 min for 64 waveform pairs.

**Figure 6:**
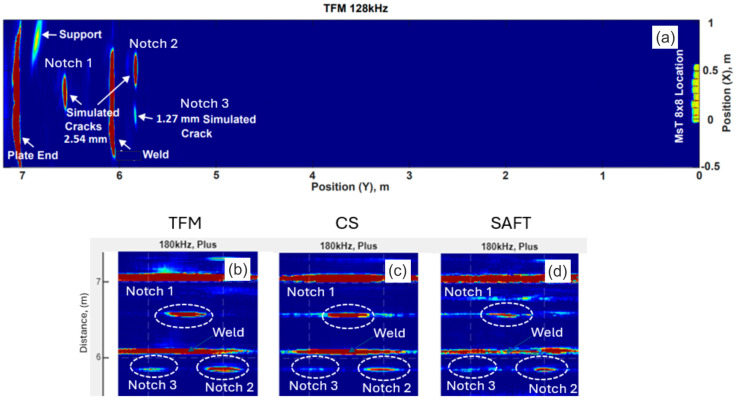
Example of anomaly imaging in a mockup plate using the TFM, CSM, and SAFT at 180 kHz: (**a**)—TFM results over the full plate; (**b**)—TFM image of the area with notches; (**c**)—CSM image of the area with notches; (**d**)—SAFT image of the area with notches.

Figure 6a shows an example of a reconstructed image of anomalies in a mockup plate using these three methods. The MsT 8 × 8 probe was mounted on the edge of a 6.4 mm thick carbon steel plate at a distance of 6 m away from target anomalies. The objective of this test was to demonstrate the ability of the probe and processing algorithm to detect and locate anomalies at a long distance from the sensor. Three EDM notches were introduced in the plate, with depths of 1.3 mm and 2.5 mm. Figure 6a shows that all three anomalies could be clearly localized from a distance of more than 6 m using the TFM, including one 2.5 mm deep notch positioned past a butt weld. The test frequency was 180 kHz. Differences in anomaly imaging using the TFM, CSM, and SAFT are presented in Figure 6. In summary, the TFM image produced the best anomaly localization and better detection of the smallest anomaly—the 1.3 mm deep notch.

**Figure 7:**
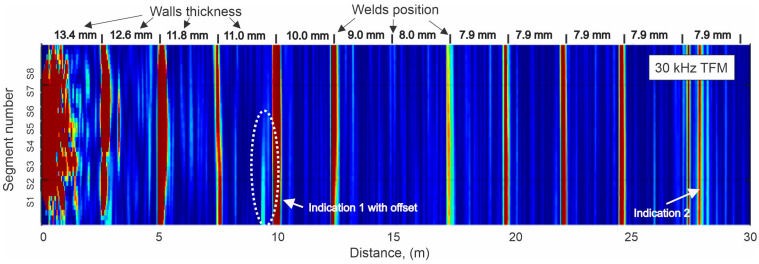
TFM image in storage tank wall obtained at 30 kHz with 8 × 8 probe mounted on tank bottom extension at axial position 0. The color scale represents the normalized signal amplitude, where warmer colors (red/yellow) indicate higher reflected amplitude and cooler colors (blue) indicate lower amplitude. All images use the same color scale.

Another example of long-range coverage is shown in Figure 7. The figure shows a TFM-reconstructed image of a 43 m high storage tank wall obtained at 30 kHz using an MsT 8 × 8 probe mounted on the tank bottom extension. This tank had extensive generalized corrosion—only the 30 kHz test frequency provided a good range of coverage in the wall. Multiple horizontal butt welds in the tank wall were clearly imaged up to the 30-m height. The TFM algorithm was able to provide lateral localization of defects up to 9 m from the probe (Indication 1). Past this distance, the lateral localization of the defect was difficult due to the large beam spread at relatively low frequency. However, the axial position of suspect Indication 2 could still be identified at 28 m.

More accurate anomaly mapping at greater distances would require using probes with wider segments or higher frequencies, if possible, in order to reduce beam spread.

## 5. Challenges of Tank Bottom Inspection Using Guided Waves

The most common challenges of tank bottom inspection from the exterior are:

Uneven and sometimes heavily rusted tank extension surfaces;

Small tank extension (less than 20–25 mm available to mount the probe);

Guided wave energy leaking into the vertical wall of the tank;

High attenuation of guided waves in the presence of generalized corrosion, deposits, or liners;

Great variety of tank bottom geometries (lap-welded, butt-welded or both, patches and penetrations) and the absence of tank geometry configuration documentation;

Presence of multiple areas with difficult access to the tank bottom extension due to other geometry features.

**Figure 8:**
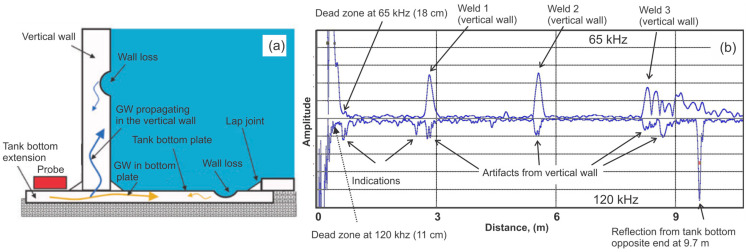
Challenges of tank bottom inspection using guided waves: (**a**)—penetration of guided waves in the tank bottom as well as in the vertical wall; (**b**)—example of data acquired from a 10 m diameter butt-welded tank bottom.

Figure 8 shows a typical tank bottom geometry with the guided wave probe mounted on the tank bottom extension, along with some sample data acquired from the butt-welded tank bottom.

Guided wave energy leaks into the vertical wall from the tank bottom because the wall is typically welded to the bottom. This results in reception of indications produced by the bottom and wall. Figure 8b shows an A-scan obtained from a 10-m-diameter storage tank with hydrofluoric acid and with a liner covering the tank bottom. Data acquired are shown as rectified; the 65 kHz waveform is shown as positive, and the 120 kHz waveform as negative. As can be noted, 120 kHz SH0 mode GWs provided full penetration through the entire bottom plate. Some artifacts produced by welds in the vertical wall could be observed at 2.8 and 5.6 m. The lower frequency provided much-more-pronounced reflections from horizontal welds in the vertical wall, but GWs at this frequency did not reach the opposite side of the tank bottom. The bottom plate of this tank was 7 mm thick and the vertical plate thickness near the tank bottom was 12.7 mm. The higher frequency performed better in the bottom, most likely because at higher frequencies, less energy is coupled to attachments [18]. It should also be noted that for the vertical wall thickness of 12.7 mm, the dead zone past the wall was only 11 cm at 120 kHz and 18 cm at 64 kHz. The dead zone might be longer or shorter depending on the thickness of the vertical plate. Indications originating from the wall can be identified by sweeping the frequency, or the 8 × 8 probe could be placed on the vertical wall at different elevations to provide GW inspection of the wall.

In the case of lap-welded tank bottoms, GW energy will perform the best in the area before the first lap weld. Some GW energy penetration occurs past the first lap weld, but the detection capability will be much lower in this region. The examples below focus on performance in lap-welded tank bottoms.

## 6. Mockup Tests

**Figure 9:**
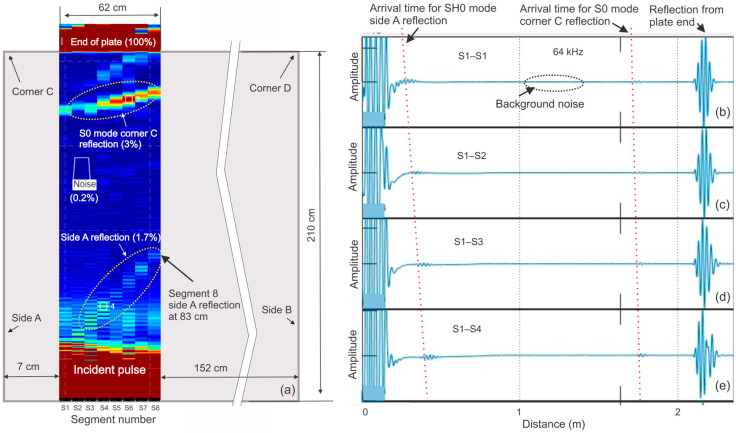
Probe testing on 260 × 210 mm × 7 mm thick carbon steel plate: (**a**)—B-scan image obtained using 8-segment probe at 64 kHz; (**b**)—A-scan obtained using segment S1; (**c**)—A-scan obtained using segments S1 and S2; (**d**)—A-scan obtained using segments S1 and S3; (**e**)—A-scan obtained using segments S1 and S4.

To determine the performance of the 8 × 8 probe, two mockups were fabricated. One mockup was a 260 × 210 × 7 mm carbon steel plate. The other mockup was a simulation of a lap-welded tank bottom with an attached vertical wall. Figure 9a shows the geometry of the first mockup with the probe positioned near the bottom edge, overlapped with the B-scan image. The B-scan consists of a sequence of A-scans acquired along a scan line, where the anomaly response is typically quantified using peak amplitude or envelope-based metrics.

The probe was intentionally offset from the bottom of the plate to capture side reflections from the near edge located 7 cm from probe segment 1. The test was conducted at 64 kHz; the resulting B-scan image was obtained using eight segments. It can be noted that side A formed a diagonal trace of SH0 mode indications corresponding to side reflections produced by segments 1–8. The apparent distance (83 cm) of the side indication produced by segment 8 is consistent with the velocity of SH0 mode waves propagating to the side at 3230 m/s. Assuming that the end of the plate was a 100% reflector, the amplitude of these side indications was 1.7%.

Another diagonal trace can be seen on the upper side of the B-scan. It has a different slope because it is from the S0 mode reflection produced by plate corner C. Based on the position of this indication, it originated from the SH0 mode wave propagating to the plate corner at 3230 m/s and returning at 5400 m/s, which is the velocity of the S0 mode for the frequency–thickness ratio (0.91) of the plate. The maximum amplitude of this set of indications was 3% of the end reflection. Figure 9b–e also show A-scans recorded using transmitter–receiver pairs of segments S1–S1, S1–S2, S1–S3, and S1–S4. Arrival times for SH0 mode side reflections and S0 mode corner reflections are marked with dotted lines. The overall accomplished SNR during this test was found to be 53.9 dB. It was calculated as the ratio between the peak reflection from the end of the plate and background noise. Compared to this number, indications produced by these side effects seem to be rather small. However, they still need to be understood to avoid false positives during field tests.

**Figure 10:**
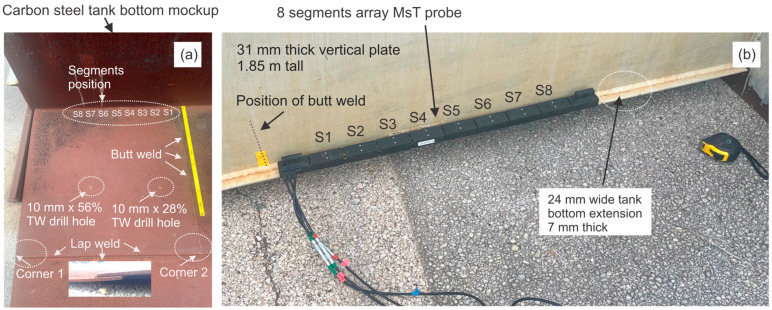
Tank bottom mockup with a lap weld: (**a**)—internal view of the mockup plate; (**b**)—external view of the mockup plate showing the position of the 8 × 8 probe.

Another mockup was fabricated to provide more realistic tank bottom conditions. The mockup consists of three 6.4 mm thick plates that comprise the tank floor and a single vertical 32 mm thick plate that represents the tank wall. The tank floor includes one lap weld that runs parallel to the tank wall and one seam weld that runs from the tank wall to the lap weld in the tank floor. The tank wall is joined to the floor by fillet welds along both edges of the vertical plate. A skirt with a width of approximately 25 mm extends beyond the wall. A photograph of the mockup portion used for the test is shown in Figure 10a. The 8 × 8 probe position is shown in Figure 10b. Two 10 mm diameter cone-bottom holes were introduced to the floor to simulate corrosion defects. The locations of both holes are highlighted with circles in Figure 10a. The depth of the holes is approximately 28% (pit 1) and 56% (pit 2) of the wall thickness. The distance to both pits from the vertical wall is 0.78 m. The probe was positioned with segment 1 located closer to a butt weld in the middle of the bottom plate as shown in Figure 10b. Pit 1 was located in the area covered by segments 1–4 and, pit 2 was located in the area covered by segments 5–8.

Conventional interpretation of guided wave test results requires a reference reflector to assign a percentage reflection to anomaly-related indications. This reference reflector could be a known geometry feature such as a weld, a plate edge or a mechanical attachment with known reflectivity. In storage tanks, the most reliable reference reflector could be the first weld in the vertical wall. However, lap welds perpendicular to the beam direction could also be used as a reference reflector. Ater assigning a percent reflection to an indication, its ranking could be based on comparison of the anomaly’s cross-section with the cross-section of a single MsT 8 × 8 segment. With a 65 mm wide probe segment, the cross-section of the area under the probe is roughly 455 mm^2^. In the case of a 10 mm diameter through-wall hole, the percent reflection of such an anomaly should be over 15%. Since the selected drill holes are 28 and 56% deep, their reflectivity should be in the range of 4 to 8%.

During reporting, the software provides tools to record the SNR value for each selected indication in reference to background noise. This information is utilized to compile indication reports. Reporting starts by assigning a reflection percent to a chosen reference reflector, and then all indications will be assigned a reflection percent based on the amplitude of the reference reflector. Ranking of indications is based on percent reflectivity values. The analysis described below is performed to find a balance between B-scan-based anomaly ranking using the indication’s peak amplitude and FMC/TFM-based ranking offering enhanced spatial resolution and a greater SNR due to integrated image energy.

**Figure 11:**
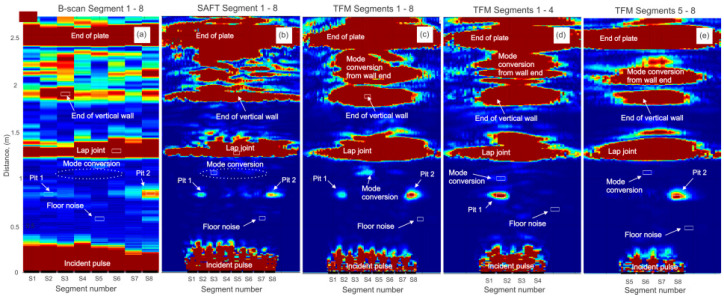
Indication reports from tank bottom mockup at 90 kHz: (**a**)—8 × 8 B-scan plot; (**b**)—8 × 8 SAFT plot; (**c**)—8 × 8 TFM plot; (**d**)—4 × 4 TFM plot using segments 1–4; (**e**)—4 × 4 TFM plot using segments 5–8.

**Figure 12:**
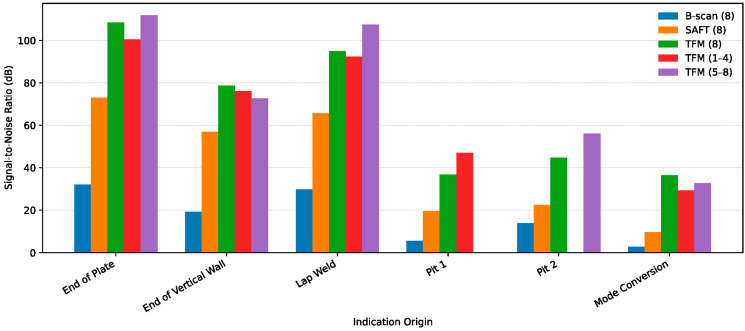
SNR for each indication shown in Figure 11.

Figure 11 shows five reconstructed images obtained at 90 kHz: an eight-segment B-scan, an eight-segment SAFT image, an eight-segment TFM image, a four-segment TFM image utilizing S1–S4, and a four-segment TFM image utilizing S5–S8. All plots were used to extract SNR information for geometry features, known defects and non-relevant (mode-converted) indications. The list of tracked indications includes the end of the bottom plate, the end of the vertical plate, a lap weld, pit 1, pit 2, and a mode-converted signal artifact. SNR values were calculated with respect to the noise floor and can be found in Table 1. A clustered plot showing SNRs measured for each indication is shown in Figure 12. Based on the measured indication amplitudes, the following conclusions can be made:

The B-scan using eight segments (Figure 10a) produced a 5.5 dB SNR for pit 1 (28% deep) and a 13.8 dB SNR for pit 2 (56% deep). The SNR of the indication produced by the lap weld was 29.8 dB versus a 32 dB SNR produced by the bottom plate end. It was estimated earlier that lap weld reflectivity could be in the order of 25–35% [19]. The pit 2 amplitude was approximately 2 times greater than the pit 1 amplitude (almost equal to a depth ratio of two). The mode-converted signal had an SNR of 2.8 dB. This level was not much greater than the noise floor and was thus unlikely to result in a false positive call. The top end of the vertical wall produced 19.3 dB versus 29.8 dB produced by the lap weld. In case of this mockup, the reflectivity from the end of the vertical wall varied a lot due to the quality of the fillet weld, and this particular reflector was not used as a reference.

Assuming the lap weld is a 35% reference reflector, pit 1 will receive reflectivity of 2.2% and pit 2 will receive reflectivity of 5.5%. Pit indication widths could be estimated with essential error (over 300%).

An eight-segment SAFT image (Figure 11b) showed a 19.5 dB SNR for pit 1 and 22.5 dB SNR for pit 2. The signal produced by mode conversion had a 9.6 dB SNR. Thus, pit 1 was detected at a 9.9 dB greater SNR than the non-defect signal. The lap weld SNR was 65.8 dB compared to the 73 dB amplitude produced by the bottom plate end. The top end of the vertical wall produced a 56.9 dB indication, indicating that only 15% of the energy propagating in the bottom plate leaked into the vertical wall. This number is different compared to readings from the B-scan. Pit indication widths could be estimated with accuracy of ±50%.

Assuming the lap weld is a 35% reference reflector, pit 1 will receive reflectivity of 0.17% and pit 2 will receive reflectivity of 0.24%. These numbers are pretty far from real values, which indicates that SNR values produced by the SAFT need to be analytically normalized.

An eight-segment TFM reconstruction image (Figure 11c) produced a 36.8 dB amplitude for pit 1 (28% deep) and 44.8 dB amplitude for pit 2 (56% deep). There is a significant improvement in the SNR for the pits. However, an artifact produced by the mode-converted signal was 36.5 dB, which is close to the amplitude of pit 1 and could potentially be misreported as a defect indication. The amplitude of the lap weld indication was 95 dB versus a 108.4 dB SNR of the bottom plate end. The top end of the vertical wall produced a 78.8 dB SNR. This implies that only 15% of the energy propagating in the bottom plate leaked into the vertical wall, similar to the result obtained using the SAFT. Pit indication widths could be estimated with accuracy of ±50%, also similar to the SAFT results. The TFM image produced about 6 dB improvement in the SNR for the pits. Indications from the lap weld and plate end gained about 30 dB. At the same time, the amplitude of the mode-converted indication also increased by 6 dB.

Assuming the lap weld is a 35% reference reflector, pit 1 will receive reflectivity of 0.04% and pit 2 will receive reflectivity of 0.15%. These numbers are far from real values. However, deducting a 31 dB excessive gain from SNR values produced by pits and a 45 gain from SNR values produced by the lap weld could produce more relevant reflectivity numbers.

Figure 11d shows TFM reconstruction of data acquired from segments S1–S4. Segments 1–4 aligned well with pit 1. This pit produced a 47 dB SNR versus a 29.3 dB SNR produced by mode conversion, 10 dB greater than that from the eight-segment TFM acquisition. The reason for this improvement is better alignment of firing and receiving segments with the anomaly location. Based on these results, it can be concluded that step-by-step image reconstruction using data from four segments at a time with an increment equal to one or two segments might produce a better SNR for anomaly-related indications and lower-amplitude mode conversion.

Figure 11e shows TFM reconstruction from the second subset of four segments, S5–S8. These segments were aligned well with pit 2. This pit produced a 56.2 dB SNR versus a 32.7 dB SNR produced by mode conversion, 12 dB higher than that from the eight-segment TFM reconstruction. Indication width estimates for pit 1 and pit 2 were more accurate using four-segment acquisition compared to eight-segment acquisition. Both indications look similar in width. SNR values for the plate end, lap weld and vertical wall are similar to those from eight-segment TFM acquisition.

In conclusion, the test on the mockup indicates the following:

Energy leaking from the sides of the probe did not exceed 1.7% compared to the energy propagating in the desired direction at 64 kHz. This effect could be ignored on real tank bottom extensions due to the absence of tank edges in a round tank.

The presence of the S0 mode and mode conversion in corners produced non-relevant indications of the order of 3% compared to the indications produced by the plate end. During testing on the mockup, these indications were suppressed more effectively when using the eight-segment SAFT or four-segment TFM, compared to reconstruction of the full set of eight-segment FMC data. For practical inspections, it might be useful to conduct TFM scans using a series of four segments (e.g., S1–S4, S2–S5, S3–S6, etc.) to help identify which indications are most likely to be actual defects.

For a given scan length (area before the first lap weld), four-segment TFM acquisition and processing produced better SNR and anomaly width estimates compared to the eight-segment TFM.

SAFT and TFM processing produced superior anomaly mapping information and SNRs compared to B-scan imaging. Anomalies had 30 dB greater SNRs and welds had 40 dB higher SNRs. Amplitude normalization and aperture weighting will be required to obtain physically meaningful reflectivity and ranking.

B-scan imaging produced more realistic reflectivity values for pit 1 (2.2%) and pit 2 (5.5%), assuming the lap weld is a 35% reference reflector. This implies that initial amplitude calibration should be based on B-scan readings. This calibration information should be used for ranking of indications generated by SAFT or TFM algorithms after deducting excessive SNR values. For higher accuracy, these values could be received from calibration mockups with anomalies representing different case scenarios.

## 7. Testing on Structures with Naturally Formed Corrosion

### 7.1. 600 mm OD Pipe with Naturally Formed Pitting

**Figure 13:**
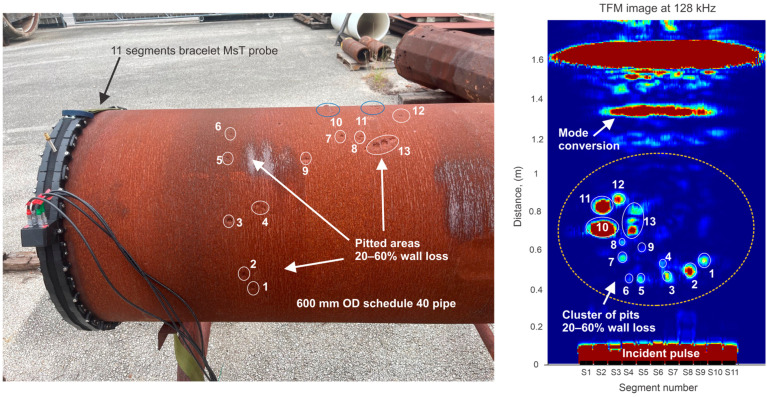
Detection of naturally formed pitting corrosion with random morphology. (**Left**)—bracelet MsT probe on the 600 mm OD pipe with pitting corrosion; (**right**)—imaging results showing detection capability after TFM processing.

The ability of the array sensor to detect naturally formed pitting was demonstrated on a section of 600 mm OD 7 mm wall pipe taken out of service. This pipe had roughly 13 pits with depths from 10 to 60% distributed in the area shown in Figure 13. An eleven-segment bracelet MsT probe was used for the test on the 250 mm OD pipe.

As seen in Figure 13, all the labeled pits in the tested sample were detected. It should be noted that the resulting image not only accurately maps the location of the pits but also produces a rough image of the pit shape. As an example, Table 2 shows the diameter and depth of pits 2, 5, 8, 11 and 12.

This information could be used to assess the cross-sectional area of the pits. If the cross-sectional area information is sufficiently accurate, then the amplitude of each indication could be correlated with the pit depth. A trace of mode-converted signal was observed near the pipe end due to the side effects described above. The location of the mode conversation trace is predictable based on the known location of the pipe end.

### 7.2. Storage Tank Walls

**Figure 14:**
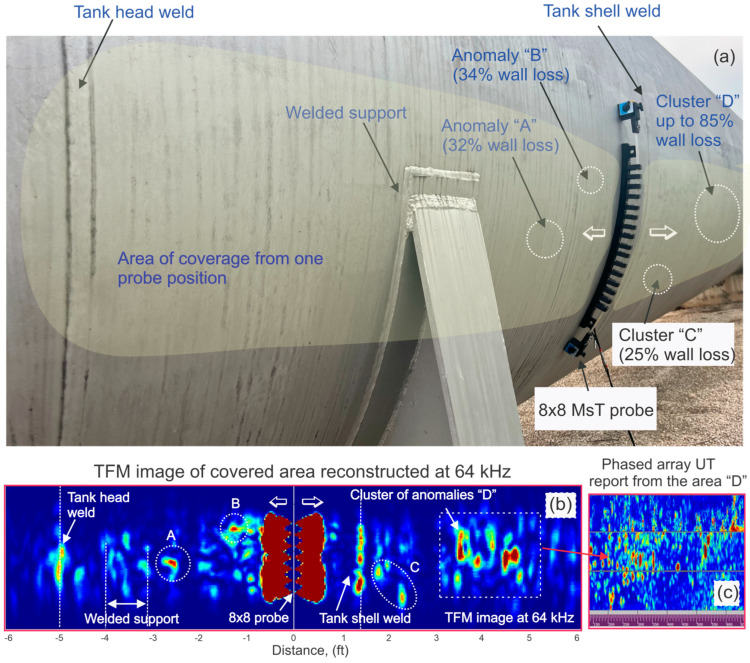
Detection of naturally formed corrosion in a storage tank shell: (**a**)—bracelet MsT probe magnetically clamped to the tank wall; (**b**)—results showing detection capability in two directions from the probe; (**c**)—results of a PA wall thickness scan of area ‘D’.

Another example includes data from a 40-year-old water storage tank with verified wall loss up to 85% in a 9.5 mm wall. Due to known extensive corrosion damage, a low frequency (60 kHz) was utilized for testing to reduce attenuation. Figure 14a shows the 8 × 8 MsT probe coupled to the tank shell. Figure 14b also shows corrosion mapping results in two directions from the probe: the negative direction covering the tank support and a cap weld and the positive direction covering a 2 m long area in the direction of the other cap.

Based on the results, the coverage range at 60 kHz was approximately 2 m from the probe in both directions. Two welds—a tank head weld and a shell weld—were clearly detected, along with four areas (A, B, C, D) that were identified as having suspected corrosion clusters. Cluster D was later mapped using a phased-array wheel probe; the resulting scan over area D is shown in Figure 14c. Based on the phased-array probe results, the most severe wall loss in the area was 85%. The corrosion morphology was rather complicated, but the location of the deepest pockets identified by the PA exam correlated well with the map produced by the MsT 8 × 8 probe. It should be noted that this region was also challenging for the PA examination, because a consistent backwall signal was not available. Also of note is that the tank support had welds on both sides; the guided wave exam covered the area under support, another somewhat challenging inspection for PA. Some minor indications were found in the area; follow-up testing would be needed to assess the actual wall loss.

The time that it takes to acquire data depends on acquisition parameters such as the number of averages, method of data collection, and acquisition length. For example, the time needed to acquire a dataset at three frequencies is 3 min for the FMC/TFM and 1 min for the SAFT or common source methods. The time for data reconstruction is once again acquisition method-, reconstruction length-, and frequency-dependent. For example, reconstruction of a 20-m image using the TFM at 30 kHz takes about 0.5 min on an 11th-generation i5-1145G7 processor with a 2.60 GHz clock. Initial reporting could be quickly performed during or right after the scan, while secondary NDE methods such as phased-array UT could be used in suspect areas on the tank wall.

### 7.3. Storage Tank Bottom

**Figure 15:**
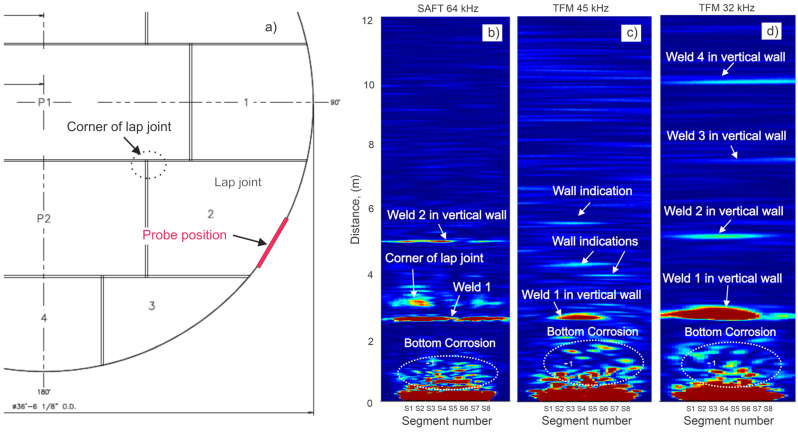
GW testing of storage tank: (**a**)—lap-welded bottom geometry and probe position; (**b**)—TFM image at 64 kHz; (**c**)—TFM image at 45 kHz; (**d**)—TFM image at 32 kHz. The color scale denotes the normalized reconstruction amplitude; red/yellow regions correspond to strong reflectors, while blue regions indicate low-amplitude background response.

The majority of storage tanks have lap-welded bottoms. Figure 15a shows the bottom scheme of a tested storage tank and the position of the 8 × 8 probe. The bottom plate was made of an 8 mm thick A36 carbon steel plate. The first two vertical wall plates were 13.4 mm and 12.6 mm thick, respectively. Due to the complexity of the tank bottom geometry and the blind zone behind the vertical wall (typically 11–20 cm), some areas were not covered. This tank was used for water storage for over 40 years; based on internal visual inspection, it had moderate-to-severe corrosion damage. Data acquisition is performed at preset frequencies (typically 30, 60, 90 and 120 kHz). Selection of the test frequency for reporting is based on criteria of the best achieved SNR and range of coverage.

Figure 15b shows a TFM-reconstructed image at a 64 kHz test frequency. The image contains indications from both the tank bottom and tank wall. Welds in the vertical wall could be clearly identified until weld 4 at a 10 m distance. A corner of the lap-welded joint in the tank bottom can be seen at 3 m. Circled areas have a number of indications likely related to corrosion. Horizontal wall scans using conventional ultrasonic testing in this area revealed the presence of only minor corrosion damage in the tank wall at this height, meaning that all moderate and severe corrosion detected by GW testing would be in the tank bottom. If minor or severe corrosion were found in the tank wall, two plots (from the tank bottom and the tank wall) would have to be overlapped to clarify the origin of the indications. Additional plots showing TFM images at 45 kHz (Figure 15c) and 32 kHz (Figure 15d) indicated that there is a cluster of significant corrosion in the tank bottom.

It should be noted that the lower frequency also resulted in a wider beam spread and larger coverage area of the bottom plate. This is the reason that the area covered by indications appears to be wider in Figure 15d compared to Figure 15b,c.

**Figure 16:**
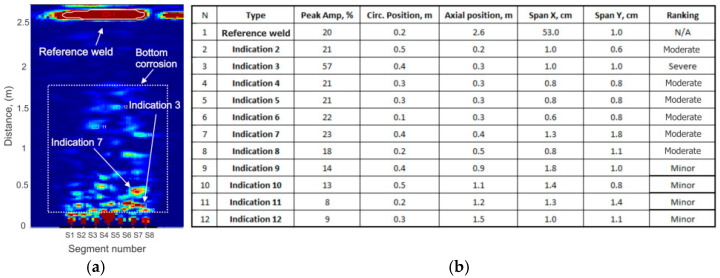
Results of anomaly ranking in tank bottom at 64 kHz: (**a**)—TFM plot showing the reference weld and area with suspect indications; (**b**)—a list of indications with relevant amplitude and position information.

Results of anomaly ranking in the area of coverage are shown in Figure 16 using a TFM image. A corrosion region marked with a dotted-line box can be seen in the 64 kHz data in Figure 16a. The first horizontal weld represents a vertical wall thickness drop from 13.4 to 12.6 mm. Together with the profile of the weld crown, the weld could be representing up to 20% cross-section change. All other indications were ranked based on indication 1 (marked as a reference weld). The maximum peak amplitude (57%) was detected at indication 3 located at a 30 cm distance from the probe. The closest to the probe indication was indication 2 located at a 20 cm distance from the probe. Three wall indications could be observed in Figure 15c at 45 kHz at a distance between 4 and 6 m. The range of coverage at 30 kHz represented in Figure 15d was limited to 12 m. The actual coverage of walls at this frequency reached 30 m, as shown in Figure 7.

Reporting software also provides approximate anomaly width (span) information. Anomaly 7 had the largest span (1.8 × 1.3 cm) and was ranked as moderate based on the amplitude information.

## 8. Conclusions

An eight-segment array of reversed-Wiedemann-effect MsT transducers was used to generate corrosion mapping in storage tank walls and bottoms using SH0 guided waves at frequencies of 30–180 kHz. The data presented in the paper addresses the side effects associated with transduction methods and contribution of unwanted S0 mode generation. The effects were assessed on mockups representing sides and a combination of butt and lap weld corners. It was shown that reducing the number of probe array segments to cover smaller areas could help reduce the amplitude of unwanted signals related to mode conversion along with simultaneously improving the SNR of anomaly-related indications. However, this approach comes at the cost of requiring the use of multiple partial-array data acquisitions to determine which signals are not anomaly-related.

An anomaly ranking method to compare reflectivity of different defects using combined B-scan- and FMC/TFM-based reports was discussed. It was shown that B-scan and FMC/TFM approaches encode SH0 scattering information differently. B-scans provide more accurate reflectivity values at the cost of lower resolution of closely spaced reflectors. TFM approaches provided dramatic improvement in anomaly detection and resolution at the cost of higher computational time and distortion of reflectivity values due to coherent summation of multiple wave packets. Therefore, amplitude normalization and reflector aperture weighting will be required to obtain physically meaningful reflectivity values.

An example of corrosion mapping was demonstrated on a 600 mm OD pipe with minor-to-moderate-size naturally formed pit clusters. Another example was a test conducted on a storage tank with a 9.5 mm wall with wide-spread generalized corrosion and with up to 85% wall loss.

An example of indication ranking in a lap-welded storage tank bottom with extensive corrosion was presented using TFM imaging. The method presented in this paper is primarily intended for screening and relative corrosion severity assessment, rather than absolute wall thickness or metal-loss quantification.

## Figures and Tables

**Table 1:** Measured SNR values for indications shown in Figure 11.

N	Type of Reflector	B-Scan 8 Segments (SNR, dB)	SAFT 8 Segments (SNR, dB)	TFM 8 Segments (SNR, dB)	TFM Segments 1–4 (SNR, dB)	TFM Segments 5–8 (SNR, dB)
1	End of Plate	32	73	108.4	100.5	111.9
2	End of Vertical Wall	19.3	56.9	78.8	76.1	72.7
3	Lap Weld	29.8	65.8	95	92.3	107.5
4	Pit 1	5.5	19.5	36.8	47	-
5	Pit 2	13.8	22.5	44.8	-	56.2
6	Mode Conversion	2.8	9.6	36.4	29.3	32.7

**Table 2:** **Geometry of selected anomalies shown on** **Figure 13.**

Anomaly No.	Diameter (mm)	Depth (%)
11	25	60
12	12	28
5	6	10
2	12	42
8	7	14

## Data Availability

The data supporting the findings of this study are not publicly available because they were generated within an internal research project and are subject to confidentiality restrictions.
